# Is It Necessary to Do Temporal Bone Computed Tomography of the Internal Auditory Canal in Tinnitus with Normal Hearing?

**DOI:** 10.1155/2013/689087

**Published:** 2013-11-26

**Authors:** Tolgar Lutfi Kumral, Guven Yıldırım, Huseyin Baki Yılmaz, Seckin Ulusoy, Guler Berkiten, Suzan Deniz Onol, Yusuf Ozturkçu, Yavuz Uyar

**Affiliations:** ^1^Department of Otorhinolaryngology, Head and Neck Surgery, Okmeydanı Training and Research Hospital, Darülaceze Caddesi, No. 25 Okmeydanı, Şişli, İstanbul 34384, Turkey; ^2^Tuzla State Hospital Otolaryngology Clinic, Istanbul, Turkey; ^3^Çorlu State Hospital Otolaryngology Clinic, Çorlu, Turkey; ^4^Department of Radiology, Okmeydanı Training and Research Hospital, İstanbul, Turkey

## Abstract

*Objective*. To investigate the compression of the vestibulocochlear nerve in the etiology of the tinnitus in the normal hearing ears with temporal bone computed tomography scans. *Methods*. A prospective nonrandomized study of 30 bilateral tinnitus and 30 normal hearing patients enrolled in this study. *Results*. A total of 60 patients (ages ranged from 16 to 87) were included. The tinnitus group comprised 11 males and 19 females (mean age 49,50 ± 12,008) and the control group comprised 6 males and 24 females (mean age 39,47 ± 12,544). Regarding the right and left internal acoustic canals measurements (inlet, midcanal, and outlet canal lengths), there were no significant differences between the measurements of the control and tinnitus groups (*P* > 0.005). There was no narrowness in the internal acoustic canal of the tinnitus group compared with the control group. High-frequency audiometric measurements of the right and left ears tinnitus group at 8000, 9000, 10000, 11200, 12500, 14000, 16000, and 18000 Hz frequencies were significantly lower than the control group thresholds (*P* < 0.05). There was high-frequency hearing loss in the tinnitus group. *Conclusion*. There were no anatomical differences in the etiology of tinnitus rather than physiological degeneration in the nerves.

## 1. Introduction

Tinnitus is the perception of sound without an external stimulus. The prevalence of tinnitus varies between 3 and 30% of all population [1]. Tinnitus is a symptom of many diseases rather than a unique disease. Nonpulsatile form is more frequent in tinnitus. Tinnitus can originate from any part of the auditory system [2].

Most of the time the etiology of nonpulsatile tinnitus is not known. Hearing loss is the most frequent known etiology [3]. Symmetric hearing loss is observed in patients with tinnitus due to noise exposure. Although tinnitus is often seen in patients with hearing loss, it can also be seen in patients with normal hearing. For this reason it is not known whether tinnitus arises from the cochlea, the hearing nerve, or the central nervous system.

The severity of tinnitus varies from mild to severe and can be bad enough to interfere with a person's daily activities even leading to distress, depression, and suicide by reducing the quality of life [4].

In order to find out the etiology of tinnitus, a good history, physical examination, radiological diagnosis, and audiological examinations are very important. Many systemic diseases such as hyperthyroidism, hypertension, and hypercholesterolemia are proven in the etiology, but the pathophysiology is still unknown [5].

As we know very well, tinnitus can be seen due to the pressure of the acoustic neuromas, cerebellopontine angle tumors, and vascular lesions, such as vascular loop to the eight cranial nerve reported in the literature. The development of the tinnitus can be observed due to nerve edema, degeneration, and compression in the canal. Accordingly, the pathological conditions that affect the width of the canal can lead to tinnitus due to compression.

This study evaluated the diameter of internal acoustic canal in physiologically impaired tinnitus patients as the etiology may be due to anatomical differences of the temporal bone.

## 2. Material and Methods

This study was performed in 30 bilateral tinnitus patients who were referred to the outpatient clinic and 30 patients without any ear disease between 2011 and 2012. Microscopic ear examination and a complete audiological examination were performed. The study group had no symptoms and signs other than tinnitus. In the physical examination, they had normal external ear canal and tympanic membrane. Patients with normal hearing thresholds in audiometric tests at the octave frequencies of 250–4,000 Hz were included in the study group. The patients with any ear complaints other than tinnitus such as chronic serous otitis media, chronic otitis media, trauma history, and external ear problems were excluded from the study. Also the patients with hyperlipidemia, hypertension, hyperthyroidism, and other systemic diseases which may cause vestibular toxicity were excluded.

**Figure 1:**
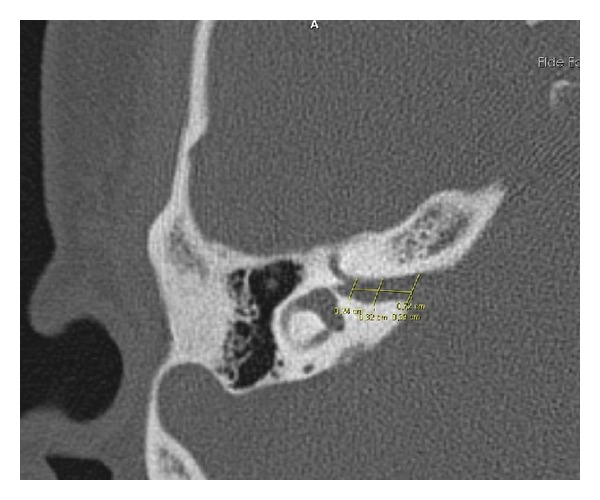
The internal auditory canal inlet, mid-canal, and outlet canal lengths were measured at the most distinctive cross-section of the seventh and eight cranial nerves bifurcation.

Both tinnitus and control groups had high-frequency audiogram at 8000, 9000, 10000, 11200, 12500, 14000, 16000, 18000 and 20000 Hz frequencies. All patients had temporal bone computed tomography imaging. The internal auditory canal inlet, mid-canal, and outlet canal lengths were measured at the most distinctive cross-section of the seventh and eighth cranial nerves bifurcation (Figure 1). Patients who were admitted to our outpatient clinic other than ear disease with the temporal bone computed tomography results were taken as control group. Informed consent and ethical approval have been taken from all the participants. Measurements of internal auditory canal inlet, mid-canal, and outlet canal lengths were compared between the groups.

### 2.1. Statistical Analysis

In the statistical model, gender (male/female), age group, measurements of internal acoustic canal, and frequencies (250, 500, 1000, 2000, 4000, 8000, 9000, 10000, 11200, 12500, 14000, 16000, 18000, and 20000 Hz) were evaluated as the main factors. For statistical analysis, SPSS 17.O V software was used to assess the findings of the study. Descriptive statistical methods (mean, standard deviation) as well as Student's *t*-test for the comparison of quantitative data showing the parameters of the normal distribution were used for the determination of difference between the groups. The significance levels were set as *P* < 0.05 and 95% confidence interval.

## 3. Results

A total of 60 patients were included in this study. The ages ranged from 16 to 87. The tinnitus group comprised 11 males and 19 females (mean age 49, 50 ± 12, 008) and control group comprised 6 males and 24 females (mean age 39, 47 ± 12, 544) (Table 1). Tinnitus and the control group did not differ significantly by gender (*P* = 0, 152) (Table 2).

Regarding the right and left internal acoustic canals measurements (inlet, mid-canal, and outlet canal length), there were no significant differences between the measurements of the control and tinnitus groups (*P* > 0.005) (Table 3).

Tinnitus group was evaluated according to internal acoustic canal measurements and there was no significant difference between the right and left canals (*P* > 0,05) (Table 4).

High-frequency audiometric measurements of the right and left ear tinnitus group at 8000, 9000, 10000, 11200, 12500, 14000, 16000, and 18000 Hz frequencies were significantly lower than the control group thresholds (*P* < 0,05). There was significant decrease at 20000 Hz frequency in the control group (*P* < 0,05) (Table 5).

## 4. Discussion

Every nerve fiber has an electric discharge, even at rest, where this represents the spontaneous activity of the nerve. In patients with tinnitus, there is an increase in this spontaneous activity. As a result, hyperactive cilia or hyperactive nerve fibers may appear and the nerve fibers perceive sounds that cannot be heard under normal conditions within central auditory and nonauditory structures [6, 7]. This could explain the persistence of tinnitus after total hearing amputation.

Tinnitus is classified as objective tinnitus (tinnitus can be heard) and subjective tinnitus which is perceived by the patient.

Today, the aim of the subjective tinnitus therapy is to increase the tolerance with sound enrichment or cognitive behavior therapy [8, 9]. Sound maskers, tinnitus-retraining therapy, and cognitive behavioral therapy are applied to relieve the symptoms caused by tinnitus. However, there is no exact solution in the treatment of tinnitus.

We investigate the anatomic reasons in the etiology of the tinnitus in the normal hearing ears. While the young population has usually normal hearing in the studies with tinnitus, hearing loss was found increasingly in the elderly. This also proves the nerve degeneration in the auditory pathways. But why are these not seen in everyone? So we evaluated the anatomy of the temporal bone by selecting patients without systemic diseases to find out if there was an anatomic difference in the tinnitus etiology.

Patients in the study group had bilateral tinnitus. Most of the patients cannot describe the localization, the time, the duration, and the severity of the tinnitus. Females were more in the tinnitus group, consistent with the literature. There was no statistically gender difference between the tinnitus group and selected control group accordingly (*P* > 0,05).

Vestibulocochlear nerve and the facial nerve enter the temporal bone through the internal auditory canal. The width of the internal acoustic canal varies from person to person. The etiology of tinnitus was influenced by many factors. But the pathophysiology is still unknown. Sometimes, all the electrophysiological studies of patients suffering from tinnitus are normal.

There were studies that showed marked improvement in tinnitus when the pathologies such as vascular loop, cerebellopontine angle tumors, and cholesteatoma were removed [10, 11]. So compression in the internal acoustic canal actually causes tinnitus.

Tinnitus and episodic vertigo attacks were also improved after microvascular decompression of the vascular loop in the internal auditory canal [12, 13]. This shows that nerve compression is effective in the etiology of tinnitus.

Meningiomas represent 3% to 12% of the tumors in the cerebellopontine angle and may be presented with tinnitus due to compression [14]. Exocytoses and osteomata are benign bony lesions of the auditory canal and they can also cause tinnitus [15].

In cases of transverse fractures of the temporal bone, the labyrinth is involved more frequently than in longitudinal fractures. This may cause hearing loss and tinnitus [16].

There have been attempts to establish relation between vestibulocochlear nerve compression site and the character of symptoms. But there is still no definitive data [7, 17].

For this purpose, the internal auditory canal diameters (inlet, mid-canal, and outlet canal lengths) were evaluated in this study. We investigated whether narrowness in any of these locations may be the cause of tinnitus. The results showed that there were no significant differences in the measurements of internal canal between control and tinnitus groups (*P* < 0,05). Also comparison of the measurements within the tinnitus groups did not show any significant difference in the right and left internal acoustic canals (*P* < 0,05).

Normal hearing thresholds can be seen in tinnitus. However, normal hearing thresholds do not necessarily indicate the absence of cochlear damage. The state of cochlea can be judged with audiogram. Therefore, cochlear damage in study group was evaluated with high-frequency audiogram. There was a significant decrease in high-frequency audiometry at 8000, 9000, 10000, 11200, 12500, 14000, 16000, and 18000 Hz frequencies even if normal audiograms at 500, 1000, 2000, and 4000 Hz in the tinnitus group (*P* < 0,05). This showed neural degeneration rather than an anatomic variation or nerve compression in the internal auditory canal. Temporal bone tomography of tinnitus patients with normal hearing at the speech frequencies (500, 1000, 2000, and 4000 Hz) did not give any additional information.

Temporal bone imaging allows fine depiction of labyrinth abnormalities related to neoplastic, inflammatory, ischemic, degenerative, or traumatic disorders [13, 18]. Magnetic resonance imaging is the best for soft tissue masses and intracranial evaluation. However, this study shows that, in normal hearing patients with tinnitus, temporal bone imaging did not give any valuable information regarding tinnitus.

## 5. Conclusion

As a conclusion, if there is a complaint of tinnitus in patients with normal hearing, temporal bone tomography does not give us valuable information and it is not cost-effective to perform it. However, if tinnitus is accompanied by hearing loss, there may be underlying acoustic neuromas or Meniere's disease. Therefore, a CT or MRI can be performed in all hearing loss patients. It was concluded that tinnitus occurred in patients with normal hearing due to pathophysiological degeneration other than anatomical variations in internal acoustic canal.

## Figures and Tables

**Table 1:** The mean age of the patients included in the study.

Age	Mean	Std. deviation	Std. error
Tinnitus group	49,50	12,008	2,192
Control group	39,47	12,544	2,290

**Table 2:** The distribution by gender of the patients in the tinnitus and control groups.

	Male	Female	Total
Tinnitus	11 (36,7%)	19 (63,3%)	30 (100,0%)
Control	6 (20,0%)	24 (80,0%)	30 (100,0%)

Total	17 (28,3%)	43 (71,7%)	60 (100,0%)

Pearson Chi-square = 2,052; *P* = 0,152.

**Table 3:** Comparison of measurements of the internal auditory canal of the tinnitus and control groups.

	Group	Mean	Std. deviation	Std. error mean	*t *	P*
Right canal inlet	Tinnitus	53,30	12,609	2,302	−0,333	0,741
	Control	54,37	12,235	2,234		

Right mid-canal	Tinnitus	46,67	10,933	1,996	1,067	0,291
	Control	43,93	8,800	1,607		

Right canal outlet	Tinnitus	29,03	5,055	0,923	0,510	0,612
	Control	28,00	9,879	1,804		

Right canal length	Tinnitus	80,13	14,178	2,589	−0,156	0,877
	Control	80,70	14,042	2,564		

Left canal inlet	Tinnitus	58,10	14,320	2,614	0,282	0,779
	Control	56,93	17,546	3,203		

Left mid-canal	Tinnitus	45,37	8,739	1,596	−0,794	0,431
	Control	47,13	8,496	1,551		

Left canal outlet	Tinnitus	30,73	6,253	1,142	−0,851	0,398
	Control	32,73	11,255	2,055		

Left canal length	Tinnitus	77,43	14,330	2,616	−0,823	0,414
	Control	80,60	15,453	2,821		

*Independent samples *t*-test.

**Table 4:** The difference between measurements of the right and left internal acoustic canal.

	*t *	P*
Right-left canal inlet	0,734	0,466
Right-left mid-canal	0,509	0,613
Right-left canal outlet	−1,378	0,174
Right-left canal length	−1,158	0,252

*Independent samples *t*-test.

**Table 5:** Comparison of high-frequency audiometric measurements between control and tinnitus groups.

Frequency (Hz)	Group	Mean	Std. deviation	*t *	P*
8000 Hz R	Tinnitus	33,50	8,725	2,110	0,039
	Control	29,33	6,397		
8000 Hz L	Tinnitus	34,17	8,718	2,445	0,018
	Control	28,83	8,167		

9000 Hz R	Tinnitus	41,17	12,365	2,445	0,019
	Control	35,00	6,159		
9000 Hz L	Tinnitus	43,83	12,154	2,771	0,007
	Control	36,50	6,159		

10000 Hz R	Tinnitus	51,50	17,027	2,294	0,025
	Control	43,33	9,499		
10000 Hz L	Tinnitus	52,00	15,625	2,271	0,027
	Control	44,00	11,326		

11200 Hz R	Tinnitus	57,50	13,818	2,873	0,006
	Control	48,33	10,694		
11200 Hz L	Tinnitus	58,00	14,118	3,429	0,001
	Control	47,33	9,535		

12500 Hz R	Tinnitus	67,67	15,797	3,100	0,003
	Control	56,17	12,777		
12500 Hz L	Tinnitus	67,00	13,038	3,187	0,002
	Control	56,83	11,633		

14000 Hz R	Tinnitus	82,50	13,630	5,660	0,000
	Control	63,67	12,101		
14000 Hz L	Tinnitus	79,00	10,860	4,634	0,000
	Control	65,83	11,148		

16000 Hz R	Tinnitus	98,67	11,137	8,424	0,000
	Control	75,50	10,462		
16000 Hz L	Tinnitus	98,33	8,442	8,088	0,000
	Control	75,50	12,955		

18000 Hz R	Tinnitus	102,17	7,621	3,719	0,000
	Control	90,67	15,128		
18000 Hz L	Tinnitus	99,83	7,931	2,367	0,000
	Control	91,50	17,575		

20000 Hz R	Tinnitus	99,83	8,251	−2,070	0,043
	Control	104,33	8,584		
20000 Hz L	Tinnitus	99,50	8,025	−3,270	0,002
	Control	106,00	7,358		

*Independent samples *t*-test.
